# Evaluating eHealth: Undertaking Robust International Cross-Cultural eHealth Research

**DOI:** 10.1371/journal.pmed.1000105

**Published:** 2009-09-15

**Authors:** David W. Bates, Adam Wright

**Affiliations:** 1 Division of General Internal Medicine, Brigham and Women's Hospital, Boston, Massachusetts, United States of America; 2 Department of Health Policy and Management, Harvard School of Public Health, Boston, Massachusetts, United States of America; 3 Harvard Medical School, Boston, Massachusetts, United States of America; 4 Partners Healthcare, Boston, Massachusetts, United States of America; The University of Edinburgh, United Kingdom

## Abstract

David Bates and Adam Wright discuss the opportunities and challenges of undertaking international collaborations in eHealth evaluation research, and make recommendations for moving forward.

*This is the second in a monthly series of three articles on evaluating eHealth*.

eHealth—the use of electronic tools in delivering health care [1]—is rapidly emerging as an international priority in nations at all levels of development, yet the benefits and priorities have not clearly been defined. The result is that there is an urgent need for additional research in this area. International research to evaluate the impact of eHealth would be especially helpful, and unless this begins to take place potential economies of scale may not be realized.

Recent events illustrate that the world economy is increasingly global; yet eHealth applications are generally local, regional, or, in a few instances, national. Nonetheless, enormous savings might be realized rapidly if international eHealth collaborations become more frequent, and more knowledge generation and even data interchange begin to occur.

International collaborations in delivering and evaluating eHealth present many opportunities but also very substantial challenges. In this Essay, we describe and discuss some of these opportunities and challenges, and present a few examples of successful international collaborations. We also lay out some suggestions and recommendations on the next steps in undertaking robust international cross-cultural eHealth research.

## Can Health Information Technology Improve Health Care Worldwide?

We believe that eHealth has enormous potential for improving care in all nations. Although islands of substantial progress exist, this potential remains largely unrealized globally. While some commentators have suggested that electronic records may be out of reach for developing nations, an increasing body of work shows that use of electronic tools can result in large health improvements, even in resource-poor environments [2]–[5], and the World Wide Web means that the latest information is now available anywhere there is an Internet connection, which in itself represents a huge development.

Although an increasing array of data show that health information technology (HIT) can improve the efficiency, quality, and safety of health care [6]–[8], the aggregate benefits are still debated and remain controversial [9],[10]. It is therefore clear that additional research is needed to better define the possible benefits of HIT even *within* countries, let alone internationally. It is perhaps not surprising that there has been little international research on eHealth across cultures, given that the area is in its infancy. This infancy is true for countries at all levels of development, but it is especially applicable for developing and transitional nations.

## A Lack of National Standardization

HIT has often been implemented at a very local level, such as at a practice or hospital level, without adhering to any specific standards. However, there have been some examples of HIT implementation with standardization, for example across a network, or region, or sometimes with national coordination. The Netherlands and Denmark, for example, have succeeded in providing some national coordination. Yet many problems occur for any hospital or practice that wishes to implement HIT, and the current fragmented approach of most nations requires each individual hospital or practice to surmount the problems they confront themselves.

One especially important problem is deciding what type of electronic decision support for health professionals should be delivered as part of the HIT. The choice of decision support matters because it has been shown that many of the benefits from the electronic record come as the result of the decision support [11]. The current norm is that every group moving ahead in this area has to reinvent the wheel, creating an enormous amount of rework. Furthermore, even when an organization finds a solution, different organizations are not communicating their success stories with each other.

The lack of HIT standardization within countries, and the way in which individual organizations are implementing electronic decision support systems in isolation without sharing experiences, makes eHealth collaborations across borders even more challenging. But other industries have been able to overcome the initial difficulties in implementing information technologies, and realized substantial changes in the process. Many believe it is time for health care to follow suit (for example, a recent editorial in *The Wall Street Journal*, entitled “Prescription for Change,” was subtitled “Health care has managed to avoid the information-technology revolution. But it won't for much longer” [12]). In particular, developments in networking and communications should make it possible to track both clinical and administrative health care data much more readily than has been previously possible. Tracking such data internationally will of course be difficult, given the language and cultural challenges. In addition, the digital divide and the varying international ownership of personal computers (which, for example, was only 2%–10% of persons in Latin America and the Caribbean in 2003 [13]) mean that many international eHealth efforts will involve novel leveraging of both cell phones and the Internet in developing countries.

## International Opportunities Provided by eHealth

Perhaps the most important of the opportunities is that a huge number of problems—like how to authenticate users or which drug–drug interactions really matter—have already been solved by someone, so that enabling sharing of solutions could dramatically reduce the costs of proceeding with HIT implementation, as well as improving the chances of success. As one example, organizations throughout the world are struggling with which specific medication-related electronic decision support to implement. Sets of such decision support are available for purchase from a number of vendors, but none of these sets fully meets provider needs. Yet a core set of medication-related decision support would likely be the same for all providers. The concept that a core exists for specific areas is true not only for the domain of decision support, but for many other areas of eHealth as well.

Another factor related to the concept just described that can be leveraged is that there is a huge gradient in development between the HIT implemented in many settings in developed countries, and the HIT now available in developing and transitional countries. Thus, it may be possible to take some solutions developed in affluent countries, and adapt them for lower-resource settings. In general, the costs of adapting software are far lower than developing it from scratch. Furthermore, wealthier nations are often willing to support work that will enable improvements in developing nations.

Yet another opportunity is that the larger the number of participants in a community, the higher the likelihood that a successful solution will emerge, especially in an open-source environment. The result of enabling truly global interchange around information technology solutions could be large economies of scale.

## Challenges of eHealth in an International Context

Despite the numerous opportunities for international eHealth collaboration, there are also very substantial challenges. There are an estimated 261 languages with more than 1 million native speakers [14], and many of these languages use varying alphabets. Despite recent developments in automation of translation, the large number of languages presents substantial challenges in translating decision support and in other eHealth applications. In developing countries, most eHealth activity is targeted towards health care professionals, who usually master an international vehicular language. Translation of applications to local languages also raises the issue of literacy because literacy varies substantially among providers, and this is especially an issue if information is made available to patients.

Less obvious than issues relating to language and literacy, but perhaps more profound as a challenge, are cultural and societal differences. These express themselves in countless ways, but one is that the ethos around performing research varies substantially among nations and regions. Especially within developing nations when the status quo with respect to health is clearly unacceptable, there is often great pressure to simply move ahead with eHealth innovations without evaluating them—yet because resources are most limited in such settings, evaluation is arguably even more important than in high-income settings. Societal issues—for example, attitudes about domestic violence—are also important and will, for example, impact the ability to identify patients for research. Specific problems will occur when last names are very frequent or often misspelled, and when dates of birth are not well recorded.

A related issue is that considerable variance exists with respect to ethics and research governance among and within nations. Elwyn et al. reported that this variance added 150 days to a multinational study protocol [15], and comparisons among European countries alone have identified substantial variation [16].

Many other issues can pose challenges for international eHealth collaboration and evaluation. There are often dramatic differences between countries in clinical systems, in how health systems are organized, and in clinical workflow. These differences make implementing similar eHealth interventions in different systems difficult and can clearly affect outcomes. Financial analyses are in many ways even more complex because of differences in care systems and structuring of health care reimbursement.

Yet another issue is that most funding for eHealth research to date has come from within countries, rather than from international sources. There are some exceptions—for example, the European Union funds international eHealth efforts, although it has elected largely to support the formation of eHealth research networks rather than research itself (if there is little or no support for research within an individual country, then the values of linkages may be modest). If truly boundary-crossing research is to be done, countries will need to relax some of their restrictions on funding moving outside national boundaries.

## Examples of Successful Collaborations

**Figure 1:**
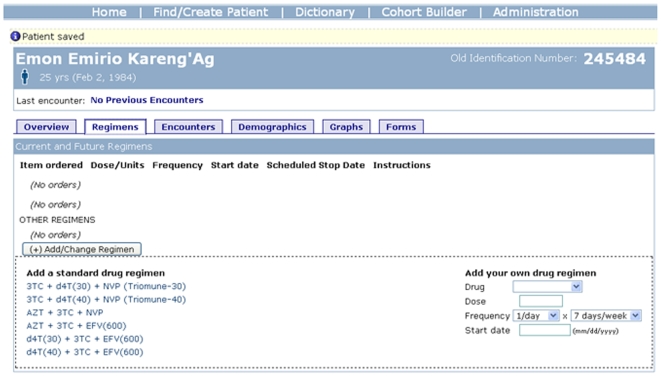
Adding an antiretroviral drug regimen for a patient with HIV in OpenMRS. (Note: The record shown here is only an example and does not represent a real patient.)

There is now a wide range of examples of international eHealth collaboration and research, such as a collaboration between the Regenstrief Institute in Indiana (http://www.regenstrief.org/) and the University of Indiana with the Moi University Faculty of Health Sciences in Kenya. This collaboration has led to the development of OpenMRS (http://openmrs.org/wiki/OpenMRS), an open-source electronic medical record designed to help track care for patients with HIV, which is now being using in a number of locations in Africa (Figure 1) [2],[3]. OpenMRS has enabled a series of retrospective studies, covering topics from assessment of outcomes for HIV-infected orphan and non-orphan children in Kenya [17], to an evaluation of the impact of an emergency plan for AIDS relief on expansion of HIV care services [18]. While much of the work on health information technologies such as OpenMRS has focused so far on development (rather than evaluation), one evaluation of OpenMRS focused on challenges in developing and maintaining a concept dictionary in a resource-poor setting, and found that most new concepts were proposed only once [19].

Other important work on eHealth has been carried out in a number of developing nations by the nonprofit international health care organization Partners in Health (http://www.pih.org/home.html). In work in Peru, this group found that use of a personal digital assistant (PDA) tool reduced data collection delays for tuberculosis laboratory results compared to paper [5]. In further work, they found that use of this application decreased work spent collecting and processing results by 60%. While the total cost and time to develop and implement the intervention was US $26,092 and 22 weeks, even more notable was that the cost to cover nine more districts was only $1,125, illustrating the potential economies of scale.

Boxes 1, 2, and 3 give further real-world examples of international eHealth collaboration. Many of these collaborations involve relationships between developing and developed nations. Among developing nations, there is great impatience to begin moving ahead rapidly on their own, for understandable reasons. In such collaborations, local autonomization is beneficial. However, since the financial resources available to support such work are severely limited in many nations, such collaborations, which are typically “north-south,” may be important for some time.

**Box 1. Integrating Cell Phones and the Internet**

A recurrent theme in international eHealth projects is the use of cell phones and the Internet together. The company Voxiva, for example, has brought these technologies together in a variety of ways, such as improving surveillance for Japanese encephalitis in Andhra Pradesh, India. The technology has enabled front-line health workers to report disease incidence through cell phones and then use analytical tools, such as geographic information systems, to understand disease prevalence [25]. Another project focused on reducing the very high maternal death rates in Ucayali, Peru; it enabled phone and Web communication between health professionals in remote areas, and then recorded all reported data in a central database [26].

**Box 2. The RAFT Network**

**Figure 2:**
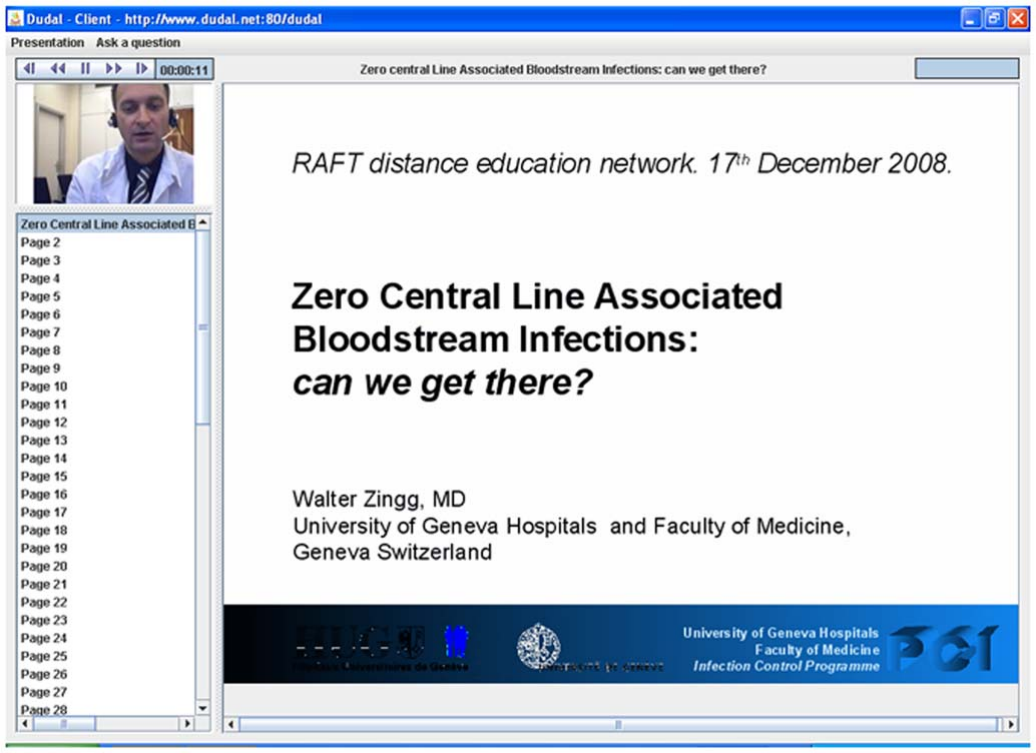
A distance continuing medical education presentation on RAFT by Walter Zingg on the subject of central line–associated infections.

The RAFT (Réseau en Afrique Francophone pour la Télémédecine) network is a collaboration between the Geneva University Hospitals and a number of countries in West Africa (see http://raft.hcuge.ch/) [27]. This network has focused on using telemedicine to enable distance continuing medical education and teleconsultations via the Internet (Figure 2).

**Box 3. Map of Medicine**

Map of Medicine is another example of an international eHealth collaboration, undertaken by a private company, which describes clinical medicine in algorithmic fashion (see http://www.mapofmedicine.com/). The map lays out “evidence-based patient journeys.” It is currently licensed to the National Health Service in England and Wales and to Queensland, Australia (http://www.mapofmedicine.com/accessthemap/accessthemap/).

## Facilitating International eHealth Evaluation

There are several ways to stimulate evaluation of international eHealth collaborations, including: (1) promoting education about the importance of conducting eHealth research, (2) developing coherence in description of eHealth interventions, (3) agreeing on common outcomes measures, and (4) improving reporting, indexing, and systematic reviewing of the literature on eHealth.

Promoting education about eHealth research is essential, especially in developing and transitional countries because of shortages of individuals with the requisite background. Furthermore, the ability to properly use scarce funding will depend on the availability of well-trained researchers.

Interventions vary tremendously in eHealth, and it can be difficult or impossible to determine what was actually done from reading a manuscript [20]. Furthermore, apparent nuances can have an important impact on the actual outcome [21]. Thus, developing consensus about how to describe eHealth interventions would be a substantial advance. However, even if there is a consensus on how to describe eHealth interventions, this won't fully overcome the problem of describing the huge array of factors involved in many eHealth applications (such as the vendor, interface, and system performance among others).

A clear area of potential is agreeing on common definitions and outcome measures. Such agreement has been highly beneficial in some other domains, such as sepsis [22] and evaluation of cardiac care. Instruments for measuring outcomes in eHealth interventions also need to be developed—for example, few cross-culturally validated instruments have been developed to measure professional or patient satisfaction with new deployments.

The increasing use of reporting guidelines for health research, collected together by the EQUATOR network (http://www.equator-network.org/), has had a major impact across a number of types of research. One of the biggest benefits of the movement to improve reporting is that it has made it clear to researchers what elements to include in both study design and reporting [23]. For example, the CONSORT statement was transformative with respect to reporting of controlled trials. An analogous statement focusing on the reporting of international cross-cultural eHealth research would likely include specifics on minimum standards for describing the technology involved, how and when it was implemented, the cultures and professions included, and what countries were involved, among other factors [24].

Finally, we need better methods for indexing and reviewing the eHealth literature. The Global Health Library (http://www.who.int/ghl/en/), which provides an electronic synthesis of eHealth literature, represents one effort to improve the visibility of the “gray literature” from developing countries. A key challenge with respect to fostering international collaborations is language, as many reports may be missed when they are published in languages other than English, and English reports will not be accessible to all.

There are several organizations that would be well placed to move these four initiatives forward, including the European Union, the World Health Organization, and the International Medical Informatics Association.

## Conclusions

A great deal might be gained if robust international evaluation of eHealth goes forward. In areas outside health, tremendous improvements in efficiency of resource utilization have already been realized, and there is every reason to suspect the benefits in health from implementation of eHealth may be similar. Clearly, if this is to be achieved, numerous obstacles—only some of which have been discussed—would need to be surmounted. However, that should be possible. Already, today, the availability of medical information has been revolutionized by the Web. Twenty years ago, the only knowledge resources in many areas in the developing world were textbooks, some of which were decades old. In contrast, today it is possible to find medical knowledge using only a cell phone almost anywhere—such changes in technology are likely to transform care, both in the developing world and outside it. To enable this transformation, a wide array of research on eHealth and its benefits will be essential.

## Abbreviations

HIT: health information technology
